# Temporal changes in nasopharyngeal carriage of *Streptococcus pneumoniae* serotype 1 genotypes in healthy Gambians before and after the 7-valent pneumococcal conjugate vaccine

**DOI:** 10.7717/peerj.903

**Published:** 2015-04-30

**Authors:** Chinelo Ebruke, Anna Roca, Uzochukwu Egere, Ousainou Darboe, Philip C. Hill, Brian Greenwood, Brendan W. Wren, Richard A. Adegbola, Martin Antonio

**Affiliations:** 1 Vaccinology Theme, Medical Research Council Unit, Banjul, The Gambia; 2 Faculty of Infectious and Tropical Diseases, London School of Hygiene & Tropical Medicine, London, United Kingdom; 3 Centre for International Health, School of Medicine, University of Otago, New Zealand; 4 GlaxoSmithKline Biologicals Wavre, Belgium; 5 Microbiology and Infection Unit, Warwick Medical School, University of Warwick, Coventry, United Kingdom

**Keywords:** Invasive pneumococcal disease, Nasopharyngeal Swab, Streptococcus pneumoniae serotype 1, Nasopharyngeal carriage, 7-valent pneumococcal conjugate vaccine, Gambia, Multilocus Sequence Typing, Sequence type, ST217 hyper virulent clonal complex, Molecular epidemiology

## Abstract

Streptococcus pneumoniae serotype 1 is one of the leading causes of invasive pneumococcal disease. However, this invasive serotype is hardly found in nasopharyngeal asymptomatic carriage and therefore large epidemiological studies are needed to assess the dynamics of serotype 1 infection. Within the context of a large cluster randomized trial conducted in rural Gambia to assess the impact of PCV-7 vaccination on nasopharyngeal carriage, we present an ancillary analysis describing the prevalence of nasopharyngeal carriage of pneumococcal serotype 1 and temporal changes of its more frequent genotypes. Nasopharyngeal swabs (NPS) were collected before PCV-7 vaccination (December 2003–May 2004) and up to 30 months after PCV-7 vaccination. The post-vaccination time was divided in three periods to ensure an equal distribution of the number of samples: (1) July 2006–March 2007, (2) April 2007–March 2008 and (3) April 2008–Feb 2009. *S. pneumoniae* serotype 1 were genotyped by MLST. Serotype 1 was recovered from 87 (0.71%) of 12,319 NPS samples collected. In the pre-vaccination period, prevalence of serotype 1 was 0.47% in both study arms. In the post-vaccination periods, prevalence in the fully vaccinated villages ranged between 0.08% in period 1 and 0.165% in period 2, while prevalence in partly vaccinated villages was between 0.17% in period 3 and 1.34% in period 2. Overall, four different genotypes were obtained, with ST3081 the most prevalent (60.71%), followed by ST618 (29.76%). ST3081 was found only in post-vaccination period 2 and 3, while ST618 had disappeared in post-vaccination period 3. Distribution of these major genotypes was similar in both study arms. Emergence of ST3081 and concomitant disappearance of ST618 may suggest a change in the molecular epidemiology of pneumococcal serotype 1 in this region. This change is not likely to be associated with the introduction of PCV-7 which lacks serotype 1, as it was observed simultaneously in both study arms. Future population-based epidemiological studies will provide further evidence of substantive changes in the pneumococcal serotype 1 epidemiology and the likely mechanisms.

## Introduction

*Streptococcus pneumoniae* is usually found in the nasopharynx of healthy individuals, which is considered a necessary step preceding invasive pneumococcal disease (IPD), including pneumonia, meningitis, and bacteraemia (Gleich et al., 2000; Bogaert, De Groot & Hermans, 2004; Baker et al., 2005; Adegbola et al., 2006; Hill et al., 2006; Ahern & Raszka, 2009; Balicer et al., 2010). There are over 90 different *S. pneumoniae* serotypes of which serotype 1 is a common cause of IPD worldwide, with particular high rates of disease in sub-Saharan Africa (Hausdorff et al., 2000; Adegbola et al., 2006; Gessner, Mueller & Yaro, 2010).

As cases of invasive disease represent only a small fraction of the pneumococcal burden, there is an increasing interest on evaluating the prevalence of pneumococcal asymptomatic carriage in the nasopharynx, since this is important in understanding the dynamics of disease and transmission as well as providing a basis for assessing the impact of interventions (Bogaert, De Groot & Hermans, 2004). Interestingly, serotype 1 is rarely found in the nasopharynx of healthy individuals with prevalence always below 1%–2% (Hill et al., 2006; Laval et al., 2006; Antonio et al., 2008; Nunes et al., 2008). As a result, there are just few studies evaluating the dynamics of serotype 1 in nasopharyngeal carriage as large epidemiological studies are needed.

Although PCV-7, the first licensed pneumococcal vaccine, did not include serotype 1 in its formulation, new PCV licensed vaccines (PCV-10 and PCV-13) include this serotype and therefore understanding the dynamics of serotype 1 carriage has become a priority. Within the context of a cluster-randomized trial conducted in rural Gambia (Roca et al., 2011), we collected a large number of NPS (12, 319 samples) before and up to 30 months after starting the trial (Hill et al., 2006; Roca et al., 2011). As an ancillary study of the trial, we describe the dynamics of pneumococcal serotype 1 nasopharyngeal carriage within a period of 6 years before and after the PCV-7 trial.

## Materials and Methods

### Study design and collection of isolates

This study was an ancillary study of large pneumococcal carriage studies conducted in 21 selected villages in rural Gambia as previously described (Hill et al., 2006; Roca et al., 2011). Firstly, a pre-vaccination cross sectional survey was conducted between December 2003 and May 2004 in which NP samples were collected from subjects of all age groups (Hill et al., 2006). Following this, a single-blind, cluster-randomized (by village) trial to evaluate the impact of PCV-7 on pneumococcal carriage was conducted in the study villages (Roca et al., 2011). In one group of 11 villages, all individuals over the age of 30 months received one dose of PCV-7, whilst subjects in this age group resident in 10 control villages received one dose of serogroup C meningococcal conjugate vaccine. All children less than 30 months of age in both study groups and infants born during the course of the trial received PCV-7. The trial showed a marked decrease of nasopharyngeal carriage of vaccine type (VT) pneumococci in all age groups and both study arms, with a more marked drop in villages where the whole community had received PCV-7 (Roca et al., 2011). There was little change in the overall prevalence of NVT carriage following introduction of the vaccine (Roca et al., 2011).

NP swabs were collected as part of several ongoing studies. First, a large pre-vaccination cross-sectional survey (CSS) conducted in 2003–2004, prior to a PCV-7 randomized trial (Hill et al., 2006). Later, as part of the cluster-randomized PCV-7 trial, NPS were collected in three different cross-sectional surveys (from 4–6 months, 12–14 months and up to 30 months) (Roca et al., 2011) and as well as a longitudinal study between 4 and 30 months (unpublished data) after vaccination (Table 1). For the purposes of this analysis, post-vaccination data were shown stratified in three different time-periods defined to ensure at least 2,000 samples in each period as the number of samples were not equally distributed throughout the follow up period (Table 1).

**Table 1:** Prevalence of nasopharyngeal pneumococcal serotype 1 carriage between pre-vaccination study period and each of the post-vaccination study periods in The Gambia.

Study period	Number of NPS	Number of serotype 1 isolates (%)	*P* value
Pre-vaccination period (December 2003–May 2004)	2,746	13 (0.47)	<0.001
Post vaccination Period 1 (July 2006–March 2007)	3,986	9 (0.23)	
Post vaccination Period 2 (April 2007–March 2008)	3,469	52 (1.50)	
Post vaccination Period 3 (April 2008–Feb 2009)	2,118	13 (0.61)	
Total	12,319	87 (0.71)	

Approval for this study was obtained from the Joint Medical Research Council (MRC)/Gambia Government Ethics Committee and the Ethics Committee of the London School of Hygiene & Tropical Medicine, UK (SCC number 1032, ISRCTN 51695599). Community and individual consent was obtained from study participants and the conduct of the trial was guided by a Data Safety and Monitoring Board.

### Serotyping and multilocus sequence typing (MLST)

A total of 87 *S. pneumoniae* serotype 1 isolates obtained from NP swabs during a survey were identified by latex agglutination (Hill et al., 2006) and confirmed by molecular serotyping (Morais et al., 2007). Multilocus sequence typing was performed on viable *S. pneumoniae* serotype 1 isolates recovered after storage at −70 °C as previously described (Antonio et al., 2008).

### Data analysis

All statistical analysis were carried out in STATA (version 11; StataCorp, College Station, Texas, USA) using Chi-square tests. *p*-Values less than 0.05 were taken to indicate statistical significance. Wet season was considered from June to October each year. Sequences were edited and aligned using the Laser Gene DNA star 7.1 software. Sequence type (ST) was obtained by submission of sequences onto the MLST database website.

## Results

A total of 12,319 NP samples were collected during the study: 22.2% of which were from the pre-vaccination period and 32.4%, 28.2% and 17.2% from the post-vaccination study periods 1–3, respectively. The median age of sampled individuals was 15 years (IQR 5.9—45 years), 11 years (IQR 4.7—29 years), 11 years (IQR 5.6—27 years) and 14 years (IQR 6.7—33 years), in the pre-vaccination and post vaccination periods 1–3 respectively. The overall prevalence of *S. pneumoniae* in the pre-vaccination period was 71.78% (1,971 out of 2,746 samples). The overall prevalence of *S. pneumoniae* in the post-vaccination periods 1–3 was 47.08% (4,507 out of 9,573 samples).

The overall prevalence of *S. pneumoniae* serotype 1 was 0.71% (87 of 12,319 samples collected). Prevalence of serotype 1 carriage was highest (1.02%) among children aged 5–14 years (*p* < 0.001) compared to other age groups. Serotype 1 carriage prevalence was highest in post-vaccination period 2 (1.50%) compared to the other study periods (*p* < 0.001) (Table 1).

Serotype 1 isolates were likely to be found during the wet season 0.81% (40 out of 4,909 samples collected) compared to the dry season 0.64% (47 out of 7,323 samples collected) (*p* = 0.268) but these were not statistically significant.

The overall prevalence of serotype 1 pneumococcal carriage was similar in vaccinated and control villages (0.73% vs. 0.68%; *p* = 0.703). In the pre-vaccination study period, prevalence of carriage of serotype 1 was the same in both vaccinated and control villages (0.47% each). However, the prevalence was lower in vaccinated than in control villages in post-vaccination period 1 (0.08% vs. 0.48%, *p* = 0.011), similar in vaccinated and control villages in period 2 (1.65% vs. 1.34%, *p* = 0.459) and higher in vaccinated villages in period 3 (1.16% vs. 0.17%, *p* = 0.004) (Table 2).

**Table 2:** Prevalence of nasopharyngeal pneumococcal serotype 1 carriage between control and vaccinated villages in each study period in The Gambia.

Study period	Village group	Number of NPS	Number of serotype 1 isolates (%)	*P* value
Pre-vaccination period	Control	1,271	6 (0.47)	0.992
	Vaccinated	1,475	7 (0.47)	
Post vaccination period 1	Control	1,468	7 (0.48)	0.011
	Vaccinated	2,518	2 (0.08)	
Post vaccination period 2	Control	1,711	23 (1.34)	0.459
	Vaccinated	1,758	29 (1.65)	
Post vaccination period 3	Control	1,171	2 (0.17)	0.004
	Vaccinated	947	11 (1.16)	

**Figure 1:**
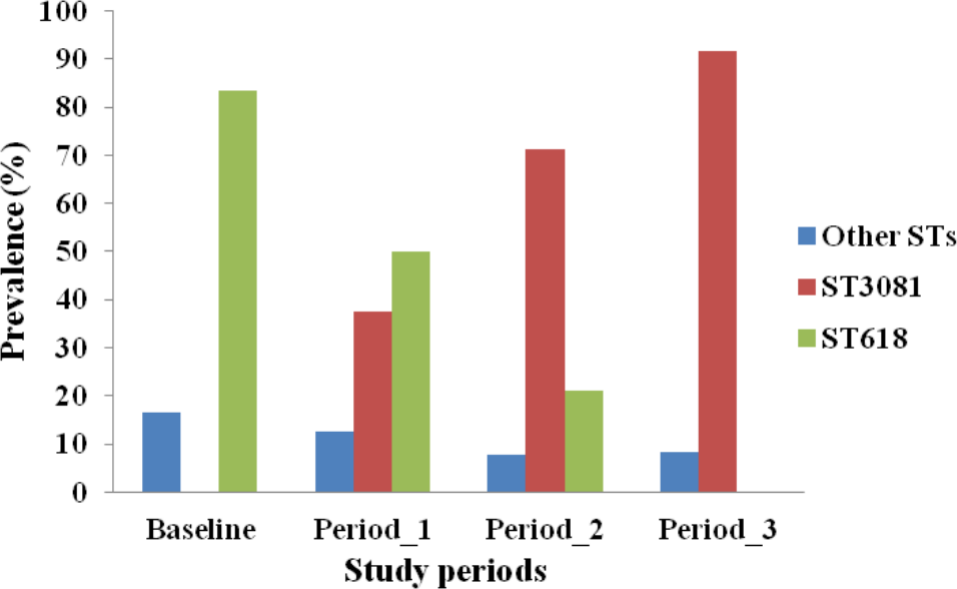
Distribution of *Streptococcus pneumoniae* 1 genotypes across study periods in The Gambia.

**Figure 2:**
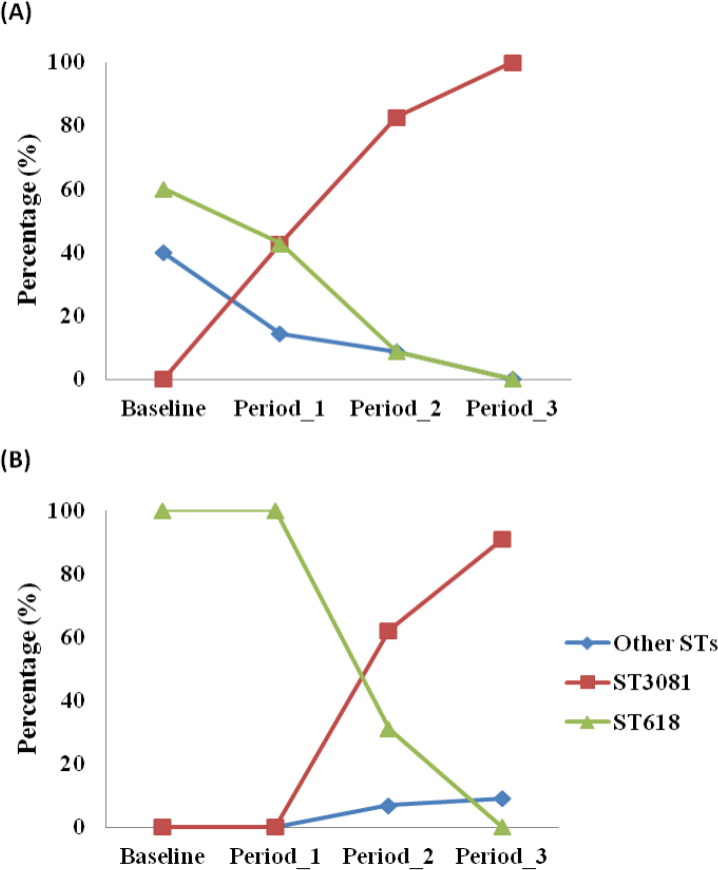
Distribution of *Streptococcus pneumoniae* 1 genotypes across study periods in (a) control and (b) vaccinated villages in The Gambia.

MLST analysis was performed for 84 of the 87 serotype 1 isolates obtained (97%). Four different STs were obtained, with ST3081 being the predominant ST (60.71%) in both vaccinated and control villages followed by ST618 (29.76%), ST217 (7.14%) and ST303 (2.38%). Prevalence of different STs was not associated with age groups (*p* = 0.368). However, the distribution of STs differed over the course of the study (*p* < 0.001). ST3081 was seen only in the post vaccination periods. ST618 was seen in the pre-vaccination and periods 1 and 2 post vaccination, but not in the post vaccination period 3 (Fig. 1). Differences in the distribution of ST over the study periods was apparent in both vaccinated (*p* = 0.002) and control (*p* = 0.021) villages (Fig. 2), with the observed expansion of ST3081 and the disappearance of ST618 occurring in both groups (Figs. 1 and 2).

## Discussion

Given that pneumococcal serotype 1 is one of the common cause of IPD worldwide and the paradox of its’ rarity in nasopharyngeal carriage, it is not unsurprising that only a few published studies have evaluated serotype 1 carriage patterns. To our knowledge, this is the largest study evaluating the dynamics of pneumococcal serotype 1 carriage. We present findings from as many as 87 serotype 1 isolates and report on the prevalence and dominant genotype patterns over a 6 year period. The finding of 0.71% overall prevalence in carriage of serotype 1 agrees with earlier findings indicating the rarity of serotype 1 in carriage studies (Brueggemann & Spratt, 2003; Hausdorff, Feikin & Klugman, 2005; Laval et al., 2006; Nunes et al., 2008; Smith-Vaughan et al., 2009). We also note that this low carriage rate was observed in both the pre- and post vaccination periods with no significant differences between study arms. However, prevalence of serotype 1 carriage was highest in the age group 5–14 years. Findings from other studies suggest that this age group is at particular risk for serotype 1 IPD as opposed to other serotypes (Adegbola et al., 2006; Gessner, Mueller & Yaro, 2010).

Introduction of the pneumococcal conjugate vaccine PCV-7 is associated with a reduction in carriage of VT serotypes but has also been linked to an increase in carriage of NVT in some settings (Mbelle et al., 1999; Huang et al., 2005; O’Brien et al., 2007) but not in our setting (Roca et al., 2011) and elsewhere (Millar et al., 2008; Roca et al., 2011). In this study, serotype 1 prevalence showed variation over the study period, but this is not likely to have been related to vaccine introduction as there was no consistent trend and no consistent difference between vaccinated and control villages. A higher carriage rate in the vaccinated group compared to the controls was observed in only one study period, and a reverse picture was observed in another post vaccination period of the study. This pattern appears more likely to be due to natural variation over time rather than to an increase in NVT serotypes due to community vaccination with PCV-7.

All STs obtained in this study belong to the ST217 hyper virulent clonal complex responsible for several epidemic outbreaks in West Africa (Leimkugel et al., 2005; Yaro et al., 2006; Antonio et al., 2008). The prevalence of the predominant serotype 1 genotypes (ST3081 and ST618) varied significantly over the study period. In period 3, we were unable to detect ST618, but noted instead the predominance of its quadruple locus variant ST3081. It is plausible that the changes between ST618 and ST3081 in this study population provide initial evidence of an expansion of the ST217 clonal complex. However, this finding could possibly have been due to temporal changes. On-going invasive pneumococcal disease surveillance studies in The Gambia such as IPD surveillance in the Upper River Region (Mackenzie et al., 2012) as well as the pneumococcal disease surveillance in the West Africa region consortium (M Antonio, pers. comm., 2015) will provide more answers to these questions. We have also shown, in our study area, the detection of a new sequence type in The Gambia without evidence that this was associated with vaccination with PCV-7. Such emergence of ST suggests natural variation in the molecular epidemiology of the pneumococcus that requires further evaluation. A report from Brazil of a study that looked at invasive serotype 1 isolates over 3 decades found temporal changes in pulse field gel electrophoresis subtypes and STs over time, but the effect of pneumococcal vaccination was not evaluated (Chiou et al., 2008). This should be closely monitored in The Gambia in the near future, as the wider PCV formulation (PCV-13) has recently been introduced as part of the Expanded Programme of Immunization.

However, we acknowledge some limitations with this study. Firstly, the samples in this study were of modest size and a larger sample size would have allowed for more robust analysis between the comparison groups. The modest number we got after sampling such a large population goes to support the notion that serotype 1 is rare in carriage. Obtaining a much larger sample size will therefore require very large epidemiological studies and its attendant challenges. Secondly, this study was limited to carriage isolates from the Western division of The Gambia. It is unclear if observations from this group are applicable to a more heterogeneous population. There is therefore a need for further studies in The Gambia, including population-based molecular epidemiological studies assessing the distribution of these STs causing IPD and whole-genome comparisons in order to identify genetic differences that could correspond with the observed differences between otherwise highly similar strains, and such studies are currently underway (Williams et al., 2012).

## Conclusions

In conclusion, in this study we show the prevalence of pneumococcal serotype 1 carriage as well as the predominant genotypes and how they varied over the study periods, but this did not seem related to community vaccination with PCV-7. This provides important baseline data for further evaluation of nasopharyngeal carriage after PCV-13 has been introduced in The Gambia.

## Abbreviations

IPD: Invasive Pneumococcal Disease
PCV-7: 7 valent pneumococcal conjugate vaccine
NPS: Nasopharyngeal Swab
MLST: Multilocus Sequence Typing
ST: Sequence type

## Additional Information and Declarations

### DNA Deposition

The following information was supplied regarding the deposition of DNA sequences: Multilocus sequencing typing (MLST) database: http://spneumoniae.mlst.net/.

## References

[ref-1] Adegbola RA, Hill PC, Secka O, Ikumapayi UN, Lahai G, Greenwood BM, Corrah T (2006). Serotype and antimicrobial susceptibility patterns of isolates of Streptococcus pneumoniae causing invasive disease in The Gambia 1996–2003. Tropical Medicine & International Health.

[ref-2] Ahern JW, Raszka WV (2009). Meningitis from an uncommon serotype of Streptococcus pneumoniae in a young child. Southern Medical Journal.

[ref-3] Antonio M, Hakeem I, Awine T, Secka O, Sankareh K, Nsekpong D, Lahai G, Akisanya A, Egere U, Enwere G, Zaman SM, Hill PC, Corrah T, Cutts F, Greenwood BM, Adegbola RA (2008). Seasonality and outbreak of a predominant Streptococcus pneumoniae serotype 1 clone from The Gambia: expansion of ST217 hypervirulent clonal complex in West Africa. BMC Microbiology.

[ref-4] Baker CI, Barrozo CP, Ryan MA, Pearse LA, Russell KL (2005). Fatal meningitis in a previously healthy young adult caused by Streptococcus pneumoniae serotype 38: an emerging serotype?. BMC Infectious Diseases.

[ref-5] Balicer RD, Zarka S, Levine H, Klement E, Sela T, Porat N, Ash N, Dagan R (2010). Control of Streptococcus pneumoniae serotype 5 epidemic of severe pneumonia among young army recruits by mass antibiotic treatment and vaccination. Vaccine.

[ref-6] Bogaert D, De Groot R, Hermans PW (2004). Streptococcus pneumoniae colonisation: the key to pneumococcal disease. The Lancet Infectious Diseases.

[ref-7] Brueggemann AB, Spratt BG (2003). Geographic distribution and clonal diversity of Streptococcus pneumoniae serotype 1 isolates. Journal of Clinical Microbiology.

[ref-8] Chiou AC, Andrade SS, Almeida SC, Zanella RC, Andrade AL, Brandileone MC (2008). Molecular assessment of invasive Streptococcus pneumoniae serotype 1 in Brazil: evidence of clonal replacement. Journal of Medical Microbiology.

[ref-9] Gessner BD, Mueller JE, Yaro S (2010). African meningitis belt pneumococcal disease epidemiology indicates a need for an effective serotype 1 containing vaccine, including for older children and adults. BMC Infectious Diseases.

[ref-10] Gleich S, Morad Y, Echague R, Miller JR, Kornblum J, Sampson JS, Butler JC (2000). Streptococcus pneumoniae serotype 4 outbreak in a home for the aged: report and review of recent outbreaks. Infection Control and Hospital Epidemiology.

[ref-11] Hausdorff WP, Bryant J, Paradiso PR, Siber GR (2000). Which pneumococcal serogroups cause the most invasive disease: implications for conjugate vaccine formulation and use, part I. Clinical Infectious Diseases.

[ref-12] Hausdorff WP, Feikin DR, Klugman KP (2005). Epidemiological differences among pneumococcal serotypes. The Lancet Infectious Diseases.

[ref-13] Hill PC, Akisanya A, Sankareh K, Cheung YB, Saaka M, Lahai G, Greenwood BM, Adegbola RA (2006). Nasopharyngeal carriage of Streptococcus pneumoniae in Gambian villagers. Clinical Infectious Diseases.

[ref-14] Huang SS, Platt R, Rifas-Shiman SL, Pelton SI, Goldmann D, Finkelstein JA (2005). Post-PCV7 changes in colonizing pneumococcal serotypes in 16 Massachusetts communities, 2001 and 2004. Pediatrics.

[ref-15] Laval CB, de Andrade AL, Pimenta FC, de Andrade JG, de Oliveira RM, Silva SA, de Lima EC, Fabio JL, Casagrande ST, Brandileone MC (2006). Serotypes of carriage and invasive isolates of Streptococcus pneumoniae in Brazilian children in the era of pneumococcal vaccines. Clinical Microbiology and Infection.

[ref-16] Leimkugel J, Adams Forgor A, Gagneux S, Pfluger V, Flierl C, Awine E, Naegeli M, Dangy JP, Smith T, Hodgson A, Pluschke G (2005). An outbreak of serotype 1 Streptococcus pneumoniae meningitis in northern Ghana with features that are characteristic of Neisseria meningitidis meningitis epidemics. Journal of Infectious Diseases.

[ref-17] Mackenzie GA, Plumb ID, Sambou S, Saha D, Uchendu U, Akinsola B, Ikumapayi UN, Baldeh I, Usuf E, Touray K, Jasseh M, Howie SR, Wattiaux A, Lee E, Knoll MD, Levine OS, Greenwood BM, Adegbola RA, Hill PC (2012). Monitoring the introduction of pneumococcal conjugate vaccines into West Africa: design and implementation of a population-based surveillance system. PLoS Medicine.

[ref-18] Mbelle N, Huebner RE, Wasas AD, Kimura A, Chang I, Klugman KP (1999). Immunogenicity and impact on nasopharyngeal carriage of a nonavalent pneumococcal conjugate vaccine. Journal of Infectious Diseases.

[ref-19] Millar EV, Watt JP, Bronsdon MA, Dallas J, Reid R, Santosham M, O’Brien KL (2008). Indirect effect of 7-valent pneumococcal conjugate vaccine on pneumococcal colonization among unvaccinated household members. Clinical Infectious Diseases.

[ref-20] Morais L, Carvalho Mda G, Roca A, Flannery B, Mandomando I, Soriano-Gabarro M, Sigauque B, Alonso P, Beall B (2007). Sequential multiplex PCR for identifying pneumococcal capsular serotypes from South-Saharan African clinical isolates. Journal of Medical Microbiology.

[ref-21] Nunes S, Sa-Leao R, Pereira LC, Lencastre H (2008). Emergence of a serotype 1 Streptococcus pneumoniae lineage colonising healthy children in Portugal in the seven-valent conjugate vaccination era. Clinical Microbiology and Infection.

[ref-22] O’Brien KL, Millar EV, Zell ER, Bronsdon M, Weatherholtz R, Reid R, Becenti J, Kvamme S, Whitney CG, Santosham M (2007). Effect of pneumococcal conjugate vaccine on nasopharyngeal colonization among immunized and unimmunized children in a community-randomized trial. Journal of Infectious Diseases.

[ref-23] Roca A, Hill PC, Townend J, Egere U, Antonio M, Bojang A, Akisanya A, Litchfield T, Nsekpong DE, Oluwalana C, Howie SR, Greenwood B, Adegbola RA (2011). Effects of community-wide vaccination with PCV-7 on pneumococcal nasopharyngeal carriage in the gambia: a Cluster-Randomized trial. PLoS Medicine.

[ref-24] Smith-Vaughan H, Marsh R, Mackenzie G, Fisher J, Morris PS, Hare K, McCallum G, Binks M, Murphy D, Lum G, Cook H, Krause V, Jacups S, Leach AJ (2009). Age-specific cluster of cases of serotype 1 Streptococcus pneumoniae carriage in remote indigenous communities in Australia. Clinical and Vaccine Immunology.

[ref-25] Williams TM, Loman NJ, Ebruke C, Musher DM, Adegbola RA, Pallen MJ, Weinstock GM, Antonio M (2012). Genome analysis of a highly virulent serotype 1 strain of Streptococcus pneumoniae from West Africa. PLoS ONE.

[ref-26] Yaro S, Lourd M, Traore Y, Njanpop-Lafourcade BM, Sawadogo A, Sangare L, Hien A, Ouedraogo MS, Sanou O, Parent du Chatelet I, Koeck JL, Gessner BD (2006). Epidemiological and molecular characteristics of a highly lethal pneumococcal meningitis epidemic in Burkina Faso. Clinical Infectious Diseases.

